# Supplementary material for: Combining Recent Nutritional Data with Prospective Cohorts to Quantify the Impact of Modern Dietary Patterns on Disability–Adjusted Life Years: A Feasibility Study
Source: Nutrients. 2020 Mar 20;12(3):833. doi: 10.3390/nu12030833 (PMC7146619; doi:10.3390/nu12030833)
Supplement: Supplementary file 1 [file nutrients-12-00833-s001.pdf]

Diet variables available in the NRP-MONICA cohort	Corresponding variables in MenuCH study [1]	Adaptations
Meat	Red and white meat	Meat products were categorized as "red & processed" or" white" in the definition of menuCH dietary patterns [1]. Here, we used the corresponding subcategories as defined in the GloboDiet software [2].
Sausage	Processed meat	
Fish	Fish	
Salad	Soups	Soups used as a proxy for supplementary intake of vegetables
Vegetables	Vegetables	
Fruits	Fruits	Nuts were pooled with fruits in the definition of menuCH dietary patterns [1]. Here, we used the "fruits" subcategory of foods as defined in the GloboDiet software [2].
Chocolate	Chocolate	
Eggs	Eggs	
Cheese	Cheese	Milk and dairy products pooled in the definition of menuCH dietary patterns [1]. Here, we used the corresponding subcategories as defined in the GloboDiet software [2].
Milk	Milk	
Yogurt	Yogurt	
Alcohol	Alcoholic beverages	NRP-MONICA data were also dichotomized to allow matching. No or Moderate alcohol consumption, no; High alcohol consumption, yes.
Dark bread	-	No corresponding variable.

Direct matching and dichotomization possible
Matching and dichotomization possible with adaptations
No matching categories between the two studies

Table S1: Selection of a subset of variables defining dietary patterns in the menuCH study and dichotomization of these variables based on the information available in the NRP-MONICA cohort

[1] Krieger et al., Nutrients, 2018
[2] Slimani et al., Eur J Clin Nutr, 2011

NCD type	Disease	Disability weight		Disease duration	
		Value	Source	Value	Source
Cancer	Malignant neoplasms, lip oral cavity and pharynx	0,53	[1]	16,39	[2]
Cancer	Malignant neoplasms, digestive organs (oesophagus, liver and bile ducts, gallbladder, unspecificied parts of the biliary tract, pancreas)	0,53	[1]	16,39	[2]
Cancer	Malignant neoplasms, digestive organs (stomach)	0,59	[1]	16,39	[2]
Cancer	Malignant neoplasms, digestive organs (small intestine colon, rectosigmoid junction, rectum, anus and anal canal, other ill-defined)	0,30	[1]	16,39	[2]
Cancer	Malignant neoplasms, respiratory systems and intrathoracic ograns	0,54	[1]	16,39	[2]
Cancer	Malignant neoplasms, bone and articular cartilage	0,30	*	16,39	[2]
Cancer	Malignant neoplasms, skin	0,08	[1]	16,39	[2]
Cancer	Malignant neoplasms, connective and soft tissue	0,30	[1]	16,39	[2]
Cancer	Malignant neoplasms, breast and female genital organs (Breast)	0,26	[2]	16,39	[2]
Cancer	Malignant neoplasms, breast and female genital organs (Ovary or unspecified)	0,30	[1]	16,39	[2]
Cancer	Malignant neoplasms, breast and female genital organs (Vulva, cervix uteri, corpus uteri or uterus unspecified)	0,12	[1]	16,39	[2]
Cancer	Malignant neoplasms, male genital organs	0,26	[1]	16,39	[2]
Cancer	Malignant neoplasms, urinary organs	0,26	[1]	16,39	[2]
Cancer	Malignant neoplasms, eye brain and CNS	0,54	[1]	16,39	[2]
Cancer	Malignant neoplasms, endocrine glands	0,20	*	16,39	[2]
Cancer	Other neoplasms (including ill-defined, multiple sites, secondary, lymphoid and hematopoietic tissue, benign and neoplasms of unknown behavior)	0,29	[3]	16,39	[2]
Diabetes mellitus	All types of diabetes	0,20	[1]	23,19	[2]
Cardiovascular	Chronic rheumatic heart diseases, hypertensive diseases, pulmonary heart diseases, other heart diseases, diseases of arteries, arterioles and capillaries, diseases of veins and lymph vessels, other disorders of the ciruclatory system	0,18	[1]	21,84	[2]
Cardiovascular	Ischemic heart diseases	0,29	[1]	21,84	[2]
Cardiovascular	Cerebrovascular diseases (stroke)	0,61	[1]	18,79	[2]
Cardiovascular	Cerebrovascular diseases (others)	0,32	[3]	18,79	[2]
Respiratory	Chronic lower respiratory diseases (COPD or unspecified chornic bornchitis)	0,31	[1]	19,60	[2]
Respiratory	Chronic lower respiratory diseases (Asthma)	0,08	[1]	27,60	[2]
Respiratory	Lung diseases due to external agents	0,41	[3]	19,60	[2]
Respiratory	Other respiratory diseases principally affecting the interstitium, Suppurative anc necrotic conditions of lower respiratory tract, other diseases of the pleura, other diseases of the respiratory system	0,23	[3]	19,60	[2]
Liver	Alcoholic liver disease, hepatic failure, cirrhosis of the liver	0,18	[3]	15,00	*

Table S2: List of disability weights, disease durations, and their respective sources by type of disease.

Note that not all types of NCDs occurred in the NRP-MONICA cohort, so only diseases present in the cohort are listed here.

[1] May et al., BMC Med, 2015

[2] Struijk et al., PLOS ONE, 2013

[3] GBD 2016 sequelae, health states, health state lay descriptions, and disability weights (http://ghdx.healthdata.org/record/global-burden-disease-study-2016-gbd-2016-disability-weights)

* Extrapolation based on diseases of the same NCD type.

CNS: Central Nervous System; COPD: Chronic Obstructive Pulmonary Disease; GBD: Global Burden of Disease; NCD: Non-Communicable Disease.

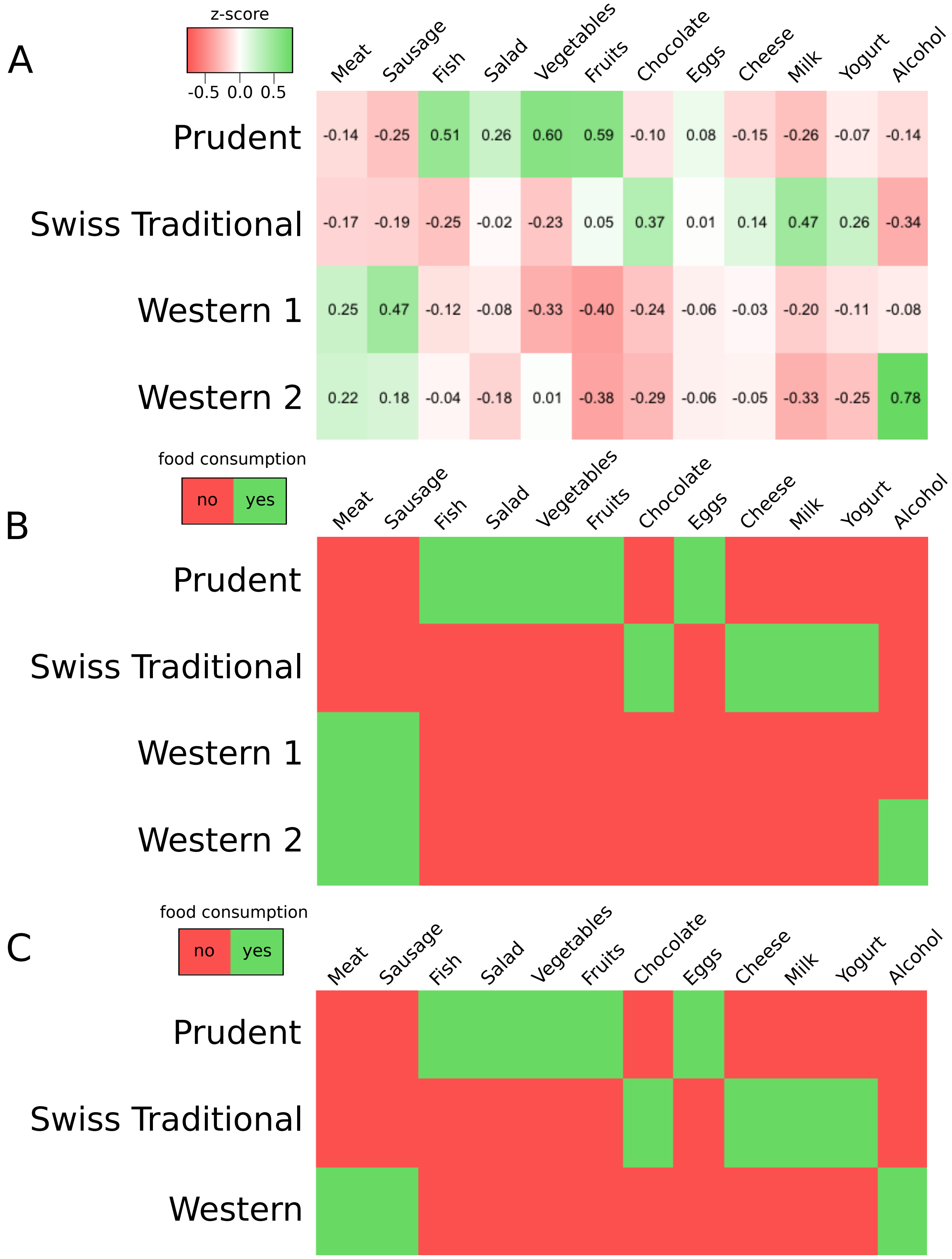

Figure S1: Dichotomization of menuCH dietary patterns.
A. Heatmap of z-scores of energy-standardized food consumption by menuCH dietary patterns. **B.** Dichotomized heatmap of of energy-standardized food consumption by menuCH dietary patterns, before and **C.** after fusion of the menuCH patterns "Western 1 (soft drinks & meat)" and "Western 2 (alcohol, meat and starchy)".

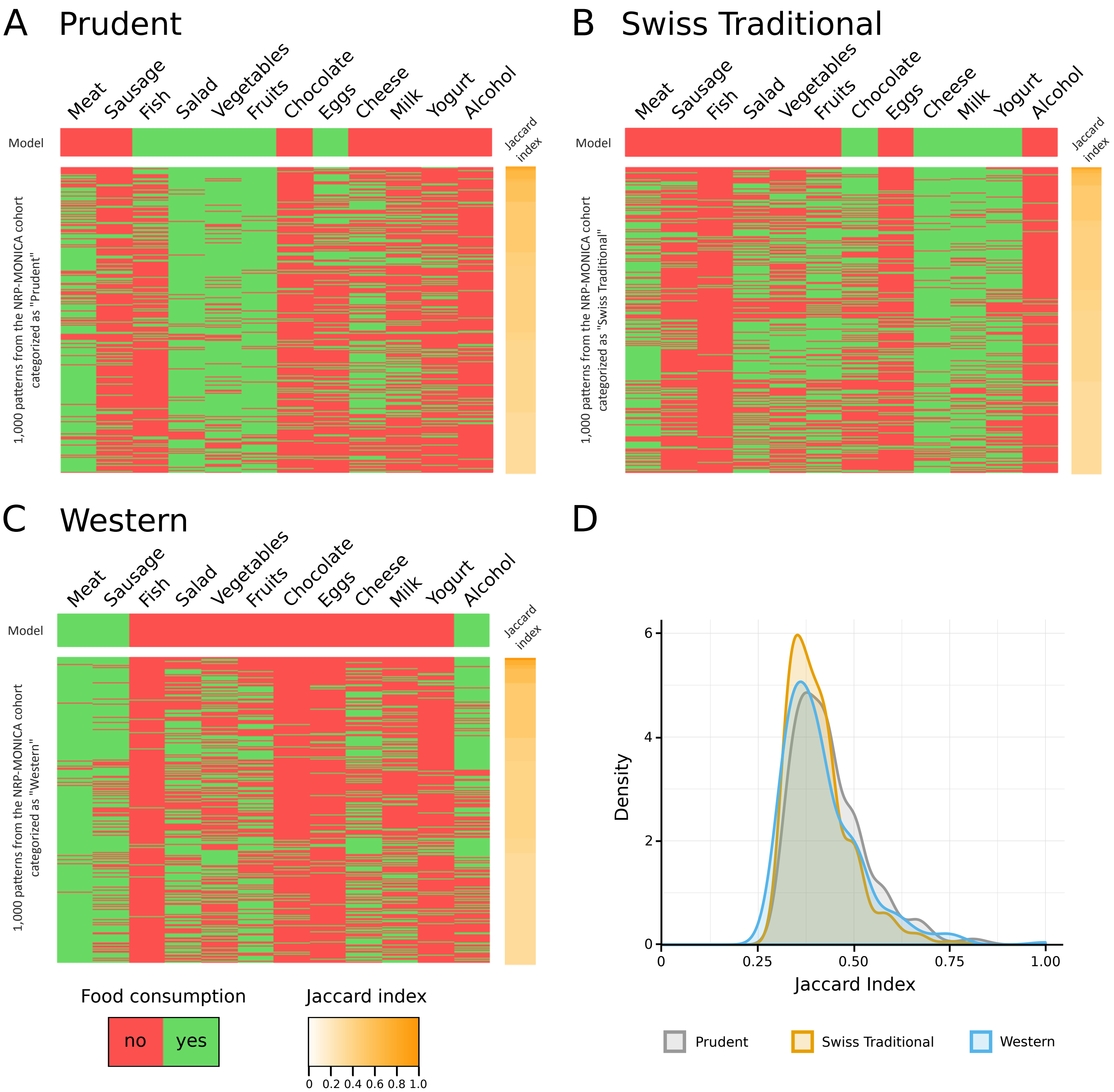

Figure S2: Similarity between menuCH modified dietary patterns and diets of participants to the NRP-MONICA cohort .

A., B., C. Each heatmap shows a random sample of 1,000 particpants' diets assigned to one of the three modified menuCH dietary patterns (dichotomized template: top bar), and ranked by Jaccard similarity index (right bar).

D. Smoothed density plot of Jaccard similarity index by dietary pattern.

A

	Prudent	Swiss Traditional	Western	Non-assigned
n	6601	2849	2508	3885
**Sum of DALYs**				
DALYs	23697	9219	10734	15121
YLL	16829	6624	7874	11021
YLD	6868	2595	2860	4100
**Mean ± SD of DALYs**				
DALYs	3.6 ± 7.8	3.2 ± 7.8	4.3 ± 8.7	3.9 ± 8.3
YLL	2.5 ± 6.1	2.3 ± 6.1	3.1 ± 6.8	2.8 ± 6.5
YLD	1.0 ± 2.3	0.9 ± 2.2	1.1 ± 2.4	1.1 ± 2.3
**DALYs-dietary patterns association**				
Crude	Reference	-0.01 [-0.28; 0.25]	**0.56 [0.24; 0.88]**	**0.27 [0.03; 0.50]**
Adjusted	Reference	-0.02 [-0.29; 0.24]	**0.30 [0.01; 0.59]**	0.15 [-0.10; 0.38]

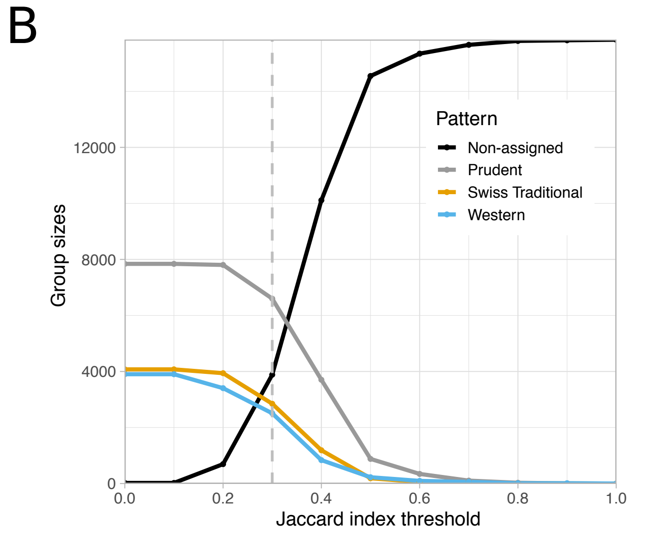

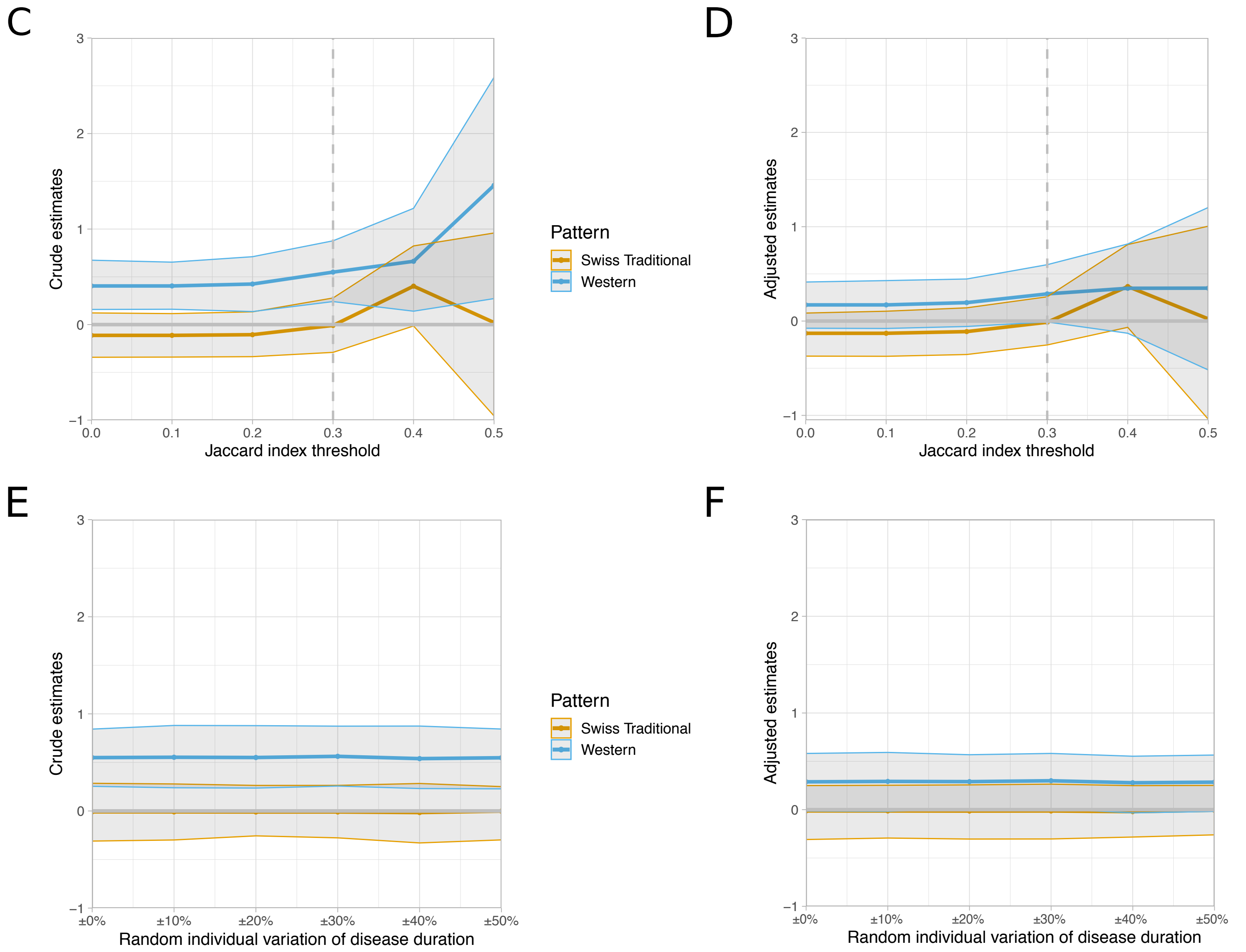

Figure S3: Sensitivity analyses.

A. DALYs due to non-communicable diseases and DALYs-dietary patterns association in the NRP-MONICA cohort when participants non-assigned to a dietary pattern are considered as a separate category. **B.** Sensitivity of group sizes to increasing thresholds of the Jaccard index. **C.** Sensitivity of the DALYs-dietary patterns association to increasing thresholds of the Jaccard index; crude and **D.** multi-adjusted estimates. **E.** Sensitivity of the DALYs-dietary patterns association to a random variation of individual disease duration; crude and **F.** multi-adjusted estimates.

DALYs: Disability-Adjusted Life Years; YLL: Years of Life Lost; YLD: Years Lost due to Disability
